# Cough sound-based estimation of vital capacity via cough peak flow using artificial neural network analysis

**DOI:** 10.1038/s41598-023-35544-3

**Published:** 2023-05-25

**Authors:** Yasutaka Umayahara, Zu Soh, Akira Furui, Kiyokazu Sekikawa, Takeshi Imura, Akira Otsuka, Toshio Tsuji

**Affiliations:** 1 Graduate School of Health Sciences, Hiroshima Cosmopolitan University, 3-2-1 Otsukahigashi, Asaminami-ku, Hiroshima Japan; 2 Graduate School of Advanced Science and Engineering, Hiroshima University, 1-4-1 Kagamiyama, Higashi-Hiroshima, Hiroshima Japan; 3 Graduate School of Biomedical and Health Sciences, Hiroshima University, 1-2-3 Kasumi, Minami-ku, Hiroshima Japan

**Keywords:** Health care, Risk factors

## Abstract

This study presents a novel approach for estimating vital capacity using cough sounds and proposes a neural network-based model that utilizes the reference vital capacity computed using the lambda-mu-sigma method, a conventional approach, and the cough peak flow computed based on the cough sound pressure level as inputs. Additionally, a simplified cough sound input model is developed, with the cough sound pressure level used directly as the input instead of the computed cough peak flow. A total of 56 samples of cough sounds and vital capacities were collected from 31 young and 25 elderly participants. Model performance was evaluated using squared errors, and statistical tests including the Friedman and Holm tests were conducted to compare the squared errors of the different models. The proposed model achieved a significantly smaller squared error (0.052 L^2^, *p* < 0.001) than the other models. Subsequently, the proposed model and the cough sound-based estimation model were used to detect whether a participant’s vital capacity was lower than the typical lower limit. The proposed model demonstrated a significantly higher area under the receiver operating characteristic curve (0.831, *p* < 0.001) than the other models. These results highlight the effectiveness of the proposed model for screening decreased vital capacity.

## Introduction

**Figure 1:**
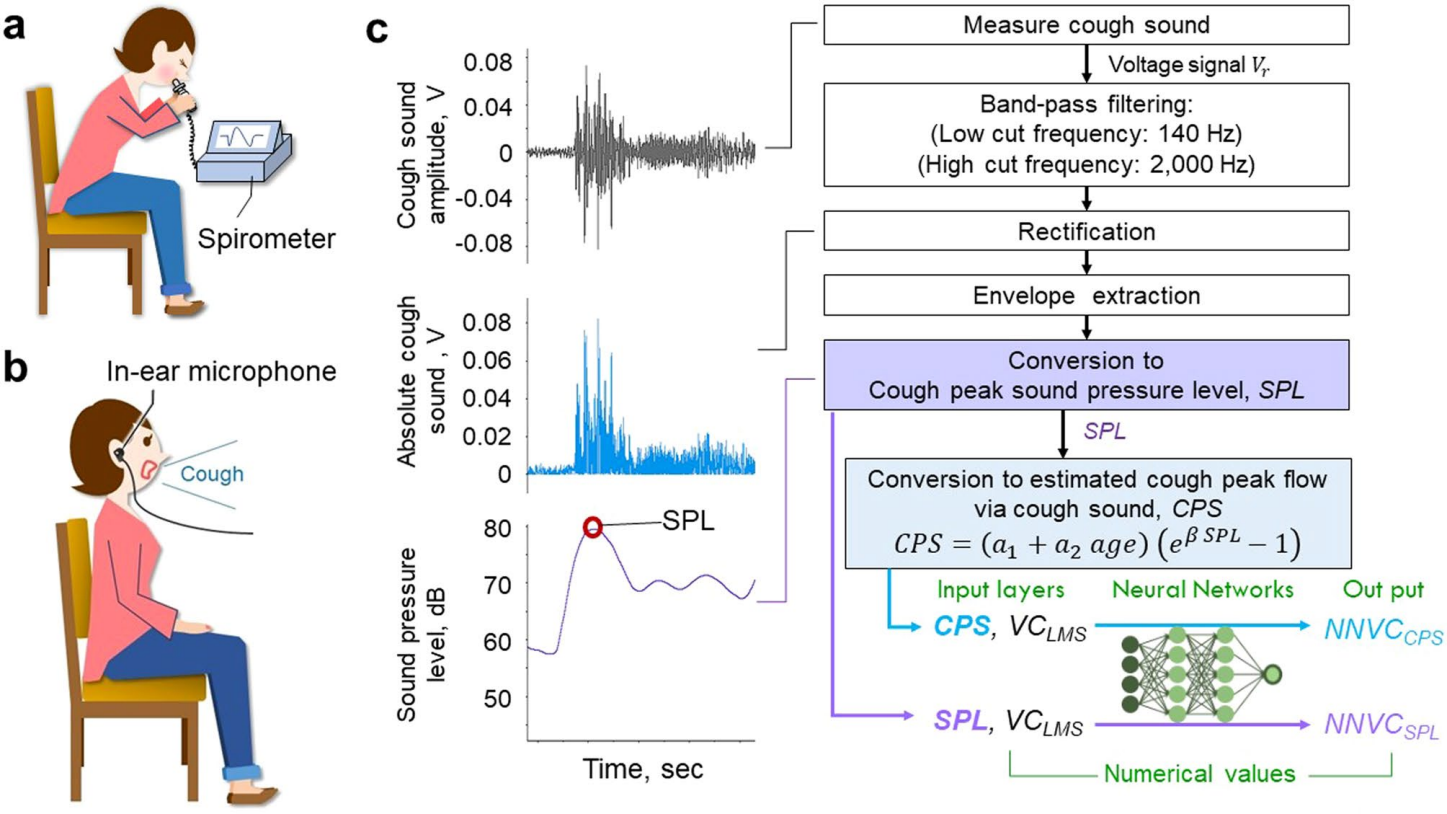
The current vital capacity measurement method and our cough sound-based method. (**a**) The current vital capacity measurement method. (**b**) The proposed vital capacity measurement method. (**c**) Data processing methods and neural networks analysis techniques for each model.

Vital capacity is a fundamental parameter used to properly interpret lung function in clinical practice. The loss of functioning lung parenchyma contributes to decreased vital capacity in many nonobstructive lung disorders^1^. Moreover, vital capacity provides prognostic information and is associated with increased mortality in the elderly population^2^. Conventionally, a spirometer is generally used (Fig. 1a) to measure vital capacity; however, this approach is expensive and inconvenient because these devices must be used in hospital settings. Thus, home-based respiratory function monitoring has attracted considerable attention^3,4^. Moreover, this vital capacity measurement method has been improved and uses a smartphone connected to a device such as a flow sensor held in the mouth by the subject^5,6^. However, a flow sensor is required for measurement and requires a mouthpiece and a filter to prevent infection, which is costly. Thus, home respiratory function monitoring would be easier and cheaper if vital capacity could be estimated without requiring a device that touches the subject’s mouth.

Vital capacity estimation has been studied for a long time. In a study published in 1948, Baldwin^7^ developed a multiple regression equation for predicting vital capacity, which depended on the individual’s characteristics, such as gender, age, and height. In 2006, the World Health Organization (WHO)^8^ deprecated the use of regression curves to predict references for biological measurements and recommended using the lambda-mu-sigma (LMS) method. This method allows simultaneous modelling of the skewness (lambda), which models the departure of the variables from normality using a Box‒Cox transformation, the mean (mu), and the coefficient of variation (sigma), for the analysis of its recently published growth standard. In 2012, the Global Lung Function Initiative announced the global lung function 2012 equations derived using the LMS method^9^. In this way, methods for calculating the reference value of vital capacity have been established and used all over the world^10–12^. The common point of each method is that aspects of the subject’s physical attributes, such as gender, age, and height, are used as explanatory variables. However, the vital capacity estimated using the LMS method (*VC*_LMS_) does not represent actual vital capacity for each individual and is merely a reference value based on the age and height of the individual^13^.

Previous studies have shown that vital capacity is related to cough strength, such as cough peak flow, which can be measured by a spirometer or peak flow metre^14,15^. The cough peak flow is an index of airway clearance ability and is related to the cough sound pressure level^16,17^, which can be easily measured by various microphones, such as condenser microphones, microphones in headsets^17^ and those built into smartphones ^15^. Moreover, the cough peak flow can be estimated via the cough sound pressure level^17,18^, which is referred to as the cough peak flow computed via cough sounds in this study. We hypothesize that the actual vital capacity of each individual could be estimated by using physical functions such as the cough peak flow computed via cough sound, which are related to both vital capacity and the subject’s physical attributes. If vital capacity can be estimated using cough sounds, flow sensors, mouthpieces, and filters would be unnecessary. More importantly, abnormal decreases in vital capacity could be detected by comparing the estimated vital capacity and the lower limit of the normal vital capacity^9^, which can be calculated by using the LMS method because pulmonary function varies with age, height, sex and ethnicity^9^.

Therefore, the purpose of this study was to estimate vital capacity using cough sounds (Fig. 1b). We employed an artificial neural network to estimate the vital capacity using the cough peak flow computed via cough sounds and *VC*_LMS_ as inputs. Because it is well known that vital capacity changes nonlinearly with age and height, we hypothesized that the artificial neural network, which uses nonlinear transformations^19^ to estimate vital capacity, could be advantageous. The estimated vital capacity was then used to detect the decrease in vital capacity below the lower limit of the normal vital capacity.

## Materials and methods

### Participants and inclusion criteria

Table 1 shows the participants’ characteristics. A total of fifty-six participants were included. Twenty-five elderly (10 male and 15 female) and 31 young (19 male and 12 female) adults participated in the experiment. The elderly participants, aged 70 to 91, lived at home where they had been receiving routine healthcare services through private arrangements. The following were exclusion criteria: a history of lung disease, institutionalization, terminal illness, unstable acute or chronic disease, a score of less than 23 on the Mini-Mental State Examination^20^, inability to give informed consent, inability to walk independently or use of a cane, and neuromusculoskeletal impairment. The young group consisted of self-reported healthy participants with no previous cardiovascular or pulmonary diseases. Participants who failed to manoeuvre the respiratory function test were excluded.

**Table 1:** Characteristics of the participants.

	Young participants (mean ± SD) *n* = 31 (Male: 19)	Elderly participants (mean ± SD) *n* = 25 (Male: 11)
Age, years	21.3 ± 0.5	80.4 ± 6.1
Height, cm	164.3 ± 8.4	154.1 ± 8.3
Weigh, kg	58.0 ± 11.5	55.7 ± 12.0
BMI, kg/m^2^	21.3 ± 0.7	23.3 ± 4.1

Values are presented as the mean ± standard deviation.

### Ethical approval

This study was conducted in accordance with the amended Declaration of Helsinki. The Hiroshima Cosmopolitan University Institutional Review Board (No. 20200305) approved the protocol, and written informed consent was obtained from all participants.

### Pulmonary function testing

Pulmonary function tests, as shown in Fig. 1a, were performed using a spirometer (Autospiro AS-507; Minato Medical Science Co., Ltd., Osaka, Japan) with the participants in a sitting position according to ATS/ERS guidelines^21^. Vital capacity was determined as the largest value from at least three acceptable manoeuvres. This measured vital capacity was utilized as the estimation target for the proposed model, as explained in subsequent sections. In addition, before the respiratory function test and cough sound measurement, an interview was conducted to check for respiratory symptoms such as acute upper respiratory tract infection or changes in physical condition. Moreover, the order of the respiratory function test and cough test was randomly assigned to minimize the effects of bias due to the measurement order, and the interval between the two tests was one week.

### Cough sound measurement system

Figures 1b,c show the cough sound measurement system. The experiment was performed as previously described^17^ using an in-ear microphone to measure cough sounds. A previous study on cough peak flow estimation via cough sound measurements reported that an in-ear microphone is suitable for measuring cough sounds due to the constant distance between the mouth of the sound source and the microphone^17^. The electret condenser microphone (in-ear microphone, ECM-TL3; Sony Corporation, Japan) was attached to the right ear canal. The measured sound signals were digitized using a 16-bit analogue-to-digital converter (PowerLab16/35, AD Instruments, Inc., Dunedin, New Zealand) at a 100 kHz sampling rate set by analysis software (LabChart version 8, AD Instruments, Inc.), and stored on a personal computer. The digitized cough sound signal was band-pass filtered between 140 and 2000 Hz to minimize artefacts caused by heart sounds and muscle interference (see Fig. 1c).

### Cough sound measurement protocols

Following thorough instructions on the coughing method provided to participants, three trials of maximal voluntary coughing were performed during each 20-s measurement period. Adequate rest periods were provided between each trial to minimize the potential impact of fatigue.

### Feature extraction

A respiratory physiotherapist with expertise in respiratory diseases extracted a 5-s segment of cough sound from each 20-s measurement period. The selected 5-s segment included the maximum cough sound in all cases. The sound pressure level, measured in dB, was subsequently determined using the following equation:

1 $$L_{P} \left( t \right) = 20\log_{10} \left\{ {\frac{{V_{r} \left( t \right)}}{{P_{0} V_{s} }}} \right\},$$

where ${V}_{r}\left(t\right)$ represents the measured voltage value, *t* is the discrete measurement time in the cough sound period, ${P}_{0}=20$ µ Pa is the reference sound pressure, and ${V}_{s}={10}^{\left(S/20\right)}$ is the voltage output per Pa. Here, $S=-35.0\mathrm{ dB }(0\mathrm{ dB }= 1\mathrm{ V}/1\mathrm{ Pa})$ is the sensitivity of the in-ear microphone. The maximum sound pressure level was calculated for each acceptable trial as follows:

2 $$L_{p,}^{\left( i \right)} = \max \left( {L_{P} \left( t \right)} \right) ,$$

where the superscript (*i*) indicates the trial. Finally, the cough sound pressure level $SPL$ was determined based on at least three acceptable trials as follows:

3 $$SPL = \max \left( {L_{{p,{\text{max}}}}^{\left( i \right)} } \right),$$

Cough peak flow is a cough strength parameter that can be estimated via cough sounds, namely, *CPS*^16,17^. Specifically, it is calculated based on the cough sound pressure level and participant age by using the following Equation^18^:

4 $$CPS = \left( {a_{1} + a_{2}\,age} \right) \left( {e^{\beta SPL} - 1} \right),$$

where ${a}_{1}, {a}_{2}$ and $\beta$ are constant parameters determined based on a nonlinear optimization scheme^18^.

### Proposed model

A previous study reported that vital capacity is related to cough strength^15^. We hypothesized that the measured vital capacity can be estimated by correcting the *VC*_LMS_ value, which reflects the height and age of a subject, using the cough peak flow computed via cough sounds. Here, *VC*_LMS_ in Liter can be estimated based on the previous literature^12^, as shown in Eqs. (5) and (6):

5 $${\text{Male}}:\,VC_{LMS} = {\text{exp}}\left( { - 8.8317 + 2.1043\,{\text{ln}}\left( h \right) - 0.1382\,{\text{ln}}\left( a \right) + m - s} \right),$$

6 $${\text{Female}}:\,VC_{LMS} = {\text{exp}}\left( { - 8.0707 + 1.9399\,{\text{ln}}\left( h \right) - 0.1678\,{\text{ln}}\left( a \right) + m - s} \right),$$

where *h* represents the participant’s height, *a* represents their age and *m-s* is the age-specific contribution from the spline function^9^.

To examine the linear relationships between the measured vital capacity and cough peak flow computed via cough sounds, partial correlation analysis was carried out. We then proposed the use of a neural network-based model to estimate the measured vital capacity from *VC*_LMS_ and the cough peak flow computed via cough sounds (see Fig. 1c and Eq. 4). To estimate the measured vital capacity, a three-layer feedforward perceptron was employed, which was composed of an input layer using an identity activation function, a hidden layer using a hyperbolic tangent function, and an output layer using an identity activation function. The number of units in the input layer was equivalent to the dimension of the input vector ${\varvec{I}}={\left[CPS, {VC}_{\mathrm{LMS}}\right]}^{T}\in {\mathbb{R}}^{2}$. The output layer, which was used to estimate the vital capacity, was configured with a single unit. The number of units in the hidden layer was set as a hyperparameter, denoted by *H*. Accordingly, the models included a total of 4*H* + 1 weight and bias parameters, which were trained using an error backpropagation algorithm. The objective of the training process was to minimize the root mean squared error, which was calculated as follows:

7 $$\sqrt {\left( {\frac{1}{N}\sum\nolimits_{i}^{N} {\left( {\widehat{{VC_{i} }} - VC_{i} } \right)^{2} } } \right)} ,$$

where ${\widehat{VC}}_{i}$ and $V{C}_{i}$ represent the estimated and measured vital capacity from observation *i*, respectively, and *N* is the total number of observations.

To determine the optimal number of units in the hidden layer, a nested cross-validation method was utilized^22^. The outer loop of this method involved training the model based on *N* − 1 observations and evaluating its accuracy based on the remaining observation. Moreover, the inner loop divided the *N* − 1 observations into two datasets, with one half of the data used to train the model using different unit numbers (*H* = 1, 2, 3) and the other half utilized to assess the estimation accuracy. This process was repeated for all possible combinations of training and test sets, and the optimal value of *H* was selected based on the highest accuracy achieved. The analyses were conducted using IBM Statistical Package for Neural Networks (SPSS) version 26.

### Verification of the estimation accuracy

To validate the efficacy of the proposed model, we compared its estimation accuracy with two different methods. First, the *VC*_LMS_ and the vital capacity estimated by the proposed model, *NNVC*_CPS_, were compared to verify the combined effectiveness of employing the neural network-based model and the cough peak flow computed via cough sounds. To evaluate the effectiveness of converting the cough sound pressure level to the cough peak flow computed via cough sounds with Eq. (4), the input vector was modified from ${\varvec{I}}={\left[CPS, {VC}_{\mathrm{LMS}}\right]}^{T}$ to ${\varvec{I}}={\left[SPL, {VC}_{\mathrm{LMS}}\right]}^{T}$. Hereafter, the vital capacity estimated based on the *SPL* and *VC*_LMS_ inputs is referred to as *NNVC*_SPL_ (see Fig. 1c). The estimation accuracy was evaluated using the mean square error $\frac{1}{N}\sum_{i}^{N}{\left(\widehat{V{C}_{i}}-V{C}_{i}\right)}^{2}$, where ${\widehat{VC}}_{i}$ and $V{C}_{i}$ represent the estimated and measured vital capacity from observation *i*, respectively, and *N* is the total number of observations, and the Spearman’s rank correlation coefficient between the measured vital capacity and the estimated vital capacities (*VC*_LMS_, *NNVC*_SPL_, and *NNVC*_CPS_). In addition, the absolute reliability of the model was investigated using regression analysis and the Bland‒Altman analysis method to detect systematic errors, such as fixed and proportional bias^23,24^. The Wilcoxon signed-rank test and, the Friedman and Holm tests^25,26^ were used for the comparison, and *p* < 0.05 was considered significant.

### Detecting abnormal decreases in vital capacity

The LLN, which can be calculated using the LMS method, represents the lower limit of the normal vital capacity, and a vital capacity less than this limit is diagnosed as respiratory dysfunction. Therefore, we detected abnormal vital capacity when the estimated vital capacities (*NNVC*_CPS_ and *NNVC*_SPL_) were less than the LNN and verified the discrimination accuracy using the area under the receiver operating characteristic^27^ curve (AUC). The AUCs resulting from *NNVC*_CPS_ and *NNVC*_SPL_ were compared using the DeLong test, where *p* < 0.05 was considered significant.

Statistical analyses other than the neural network analysis were performed with IBM SPSS version 26 and EZR (Saitama Medical Center, Jichi Medical University, Saitama, Japan)^28^, which is a graphical user interface for R (the R Foundation for Statistical Computing, Vienna, Austria).

## Results

### Relationships between vital capacity and the body structure parameters and cough peak flow

Table 2 shows that the partial correlations between the vital capacity and cough peak flow, age, and height were 0.286 (*p* = 0.038), − 0.718 (*p* < 0.001), and 0.683 (*p* < 0.001), respectively. Because there was no significant partial correlation between vital capacity and weight, we excluded this parameter from the input for the neural network-based model for estimating vital capacity.

**Table 2:** Partial correlation analysis results, *n* = 56.

	Cough peak flow (*p value*)	Age (*p* value)	Height (*p* value)	Weight (*p* value)
Vital capacity	0.286* (0.038)	− 0.718** (< 0.001)	0.683** (< 0.001)	0.002 (0.987)
Cough peak flow	–	− 0.223 (0.108)	0.037 (0.795)	0.131 (0.350)
Age	–	–	0.388** (0.004)	0.202 (0.148)
Height	–	–	–	0.406** (0.003)

The *p* values are noted in parentheses, and those less than 0.05 and 0.01 are labelled * and **, respectively.

### Vital capacity estimation accuracy

**Figure 2:**
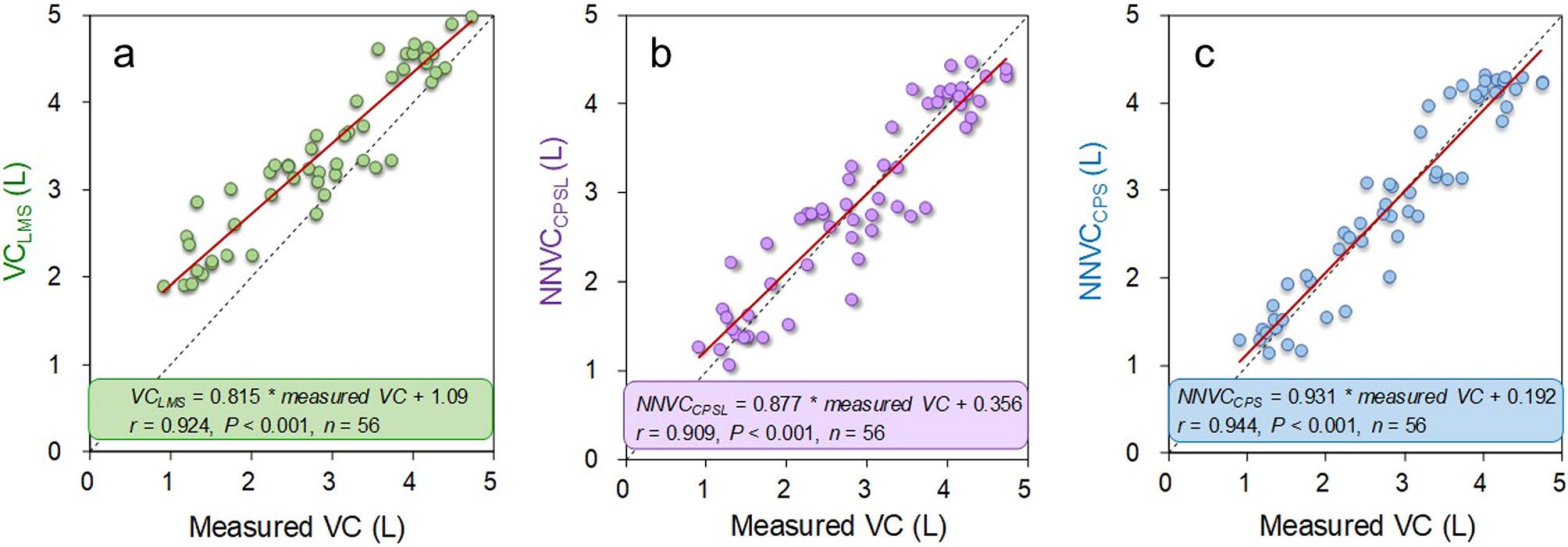
Estimation accuracy of *VC*_LMS_*, NNVC*_SPL_ and *NNVC*_CPS_. (**a**) Plot of *VC*_LMS_ versus the measured vital capacity. (**b**) Plot of *NNVC*_SPL_ versus the measured vital capacity. (**c**) Plot of *NNVC*_CPS_ versus the measured vital capacity. The linear regression lines are drawn in red, and the corresponding equations are shown in the lower part of each figure. The left lower side shows the correlation coefficients and *p* values for each scatter plot.

**Figure 3:**
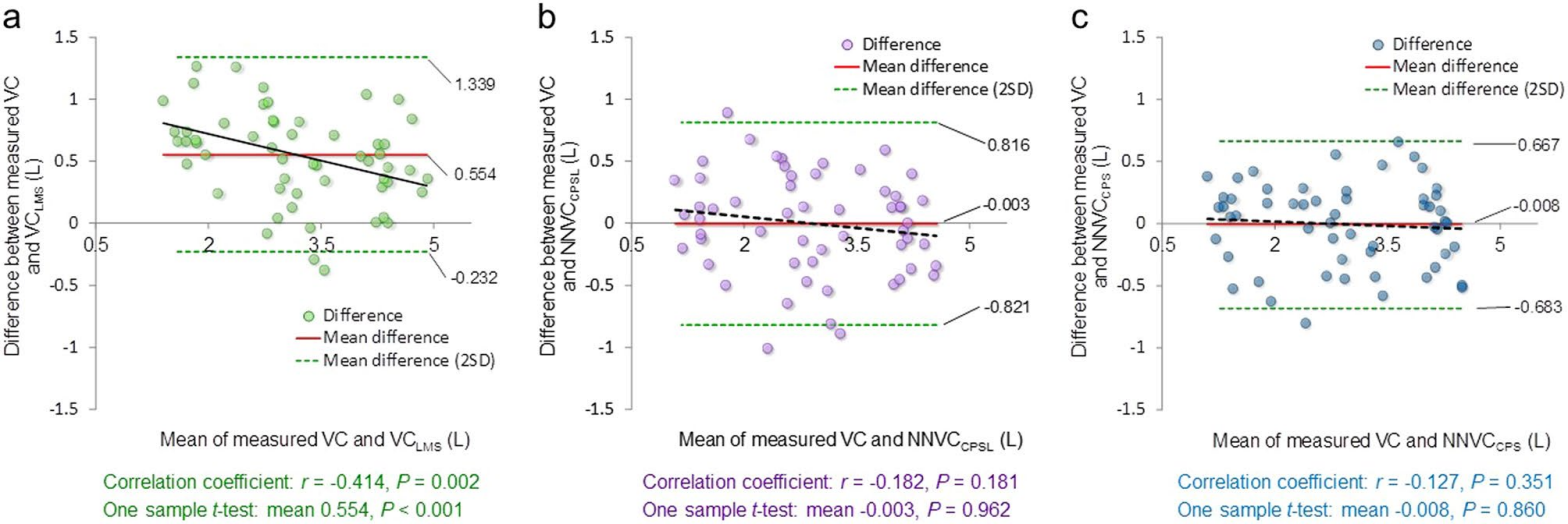
Bland‒Altman plots of the measured and estimated vital capacities. (**a**) The estimation accuracy of *VC*_LMS_. (**b**) The estimation accuracy of *NNVC*_SPL_. (**c**) The estimation accuracy of *NNVC*_CPS_. The horizontal line shows the mean of the measured and estimated vital capacities. The red line shows the mean of the measured and estimated vital capacities. The vertical line represents the difference between the measured and estimated vital capacities. The bold black solid lines represent the mean differences between the measured and estimated vital capacities, and the green dotted lines represent ± 2 standard deviations of the mean differences.

**Figure 4:**
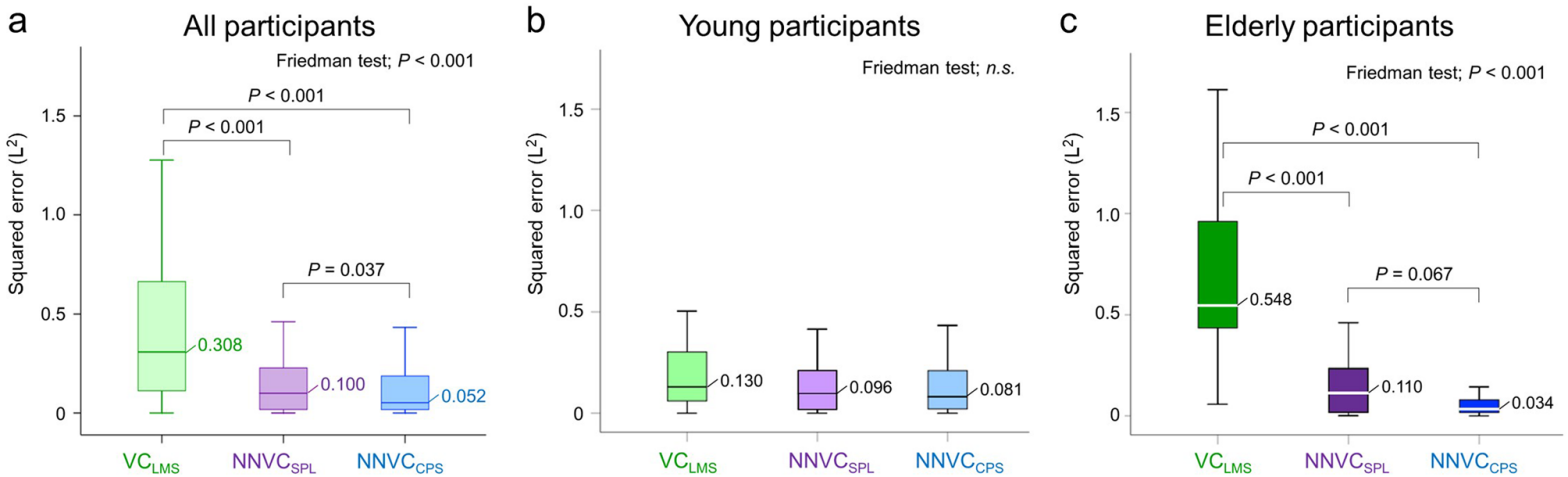
Comparison of the squared error between the previous and proposed models. (**a**) Results of all participants, *n* = 56. (**b**) Results of young participants, *n* = 31. (**c**) Results of elderly participants, *n* = 25. The *p* value was adjusted using the Holm test^25,26^ for multiple testing.

**Figure 5:**
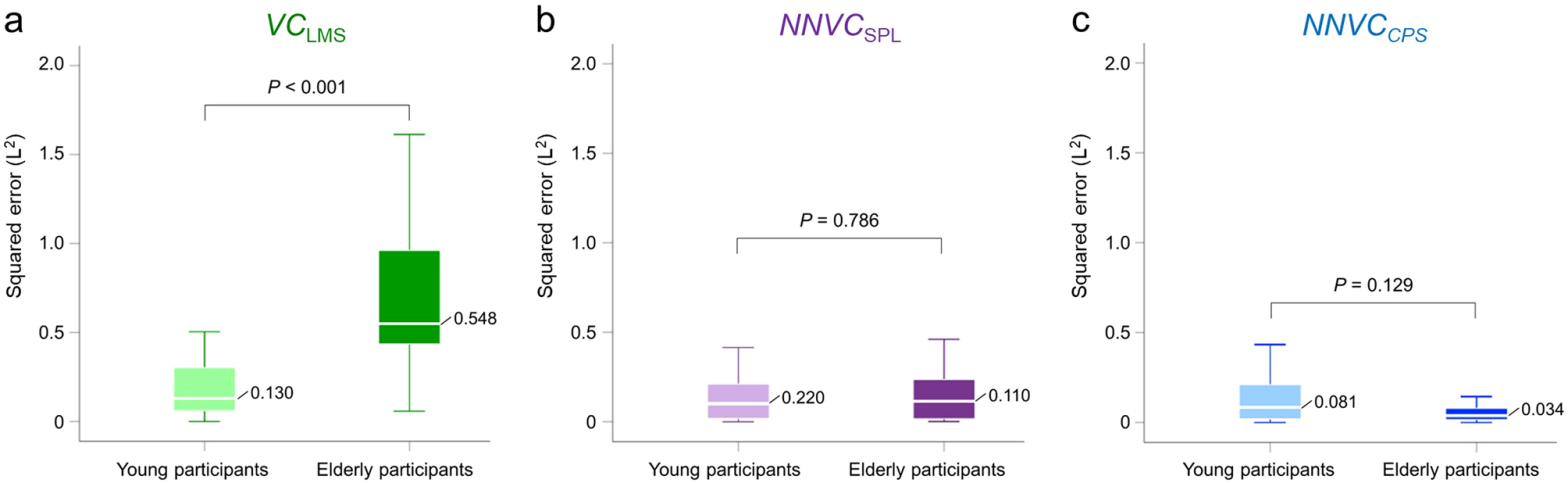
Comparison of the squared error between generations. (**a**) Results of *VC*_LMS_. (**b**) Results of *NNVC*_SPL_. (**c**) Results of *NNVC*_CPS_. Young participants, *n* = 31. Elderly participants, *n* = 25.

Leave-one-out cross-validation analysis showed that the root mean squared errors of the *NNVC*_SPL_ and *NNVC*_CPS_ against the measured vital capacity were 0.165 L and 0.112 L, respectively. Figure 2 shows the relationships between the measured vital capacity and the estimated vital capacities, indicating correlation coefficients of 0.924 (*p* < 0.001) for *VC*_LMS_, 0.909 (*p* < 0.001) for *NNVC*_SPL,_ and 0.944 (*p* < 0.001) for *NNVC*_CPS_. Figure 3 shows the corresponding Bland‒Altman plots. Neither *NNVC*_SPL_ nor *NNVC*_CPS_ showed systematic errors, but *VC*_LMS_ showed a fixed bias (one sample *t test*; *p* < 0.001) and a proportional bias (*r* = − 0.414; *p* = 0.002). Furthermore, the Friedman and Holm tests showed significant differences in the squared error between *VC*_LMS_ and *NNVC*_SPL_, *VC*_LMS_ and *NNVC*_CPS,_ *NNVC*_SPL_ and *NNVC*_CPS_ (median 0.308 L^2^ vs. 0.100 L; *p* = 0.001, 0.308 L^2^ vs. 0.052 L; *p* < 0.001, 0.100 L^2^ vs. 0.052 L^2^; *p* = 0.037, respectively) (see Fig. 4a). In young participants, the Friedman test showed no significant differences in the squared error (*p* = 0.198) (see Fig. 4b); however, among elderly participants, the Freidman and Holm tests showed significant differences in the squared error between the *VC*_LMS_ and *NNVC*_SPL_, *VC*_LMS_ and *NNVC*_CPS_ (0.548 L^2^ vs. 0.110 L^2^; *p* < 0.001, 0.548 L^2^ vs. 0.034 L^2^; *p* < 0.001, respectively) (see Fig. 4c). Figure 5 demonstrates the results of comparing the squared error between generations. The Wilcoxon signed-rank test showed significant differences in the squared error of *VC*_LMS_ between young and elderly participants (0.130 L^2^ vs. 0.548 L^2^; *p* < 0.001) (see Fig. 5a); however, there were no significant differences in the squared error for *NNVC*_SPL_ between generations (see Fig. 5b). Although there was no significant difference in the *NNVC*_CPS_ between generations, the squared error among the elderly participants was approximately 40% lower than that of the young participants (see Fig. 5c).

### Detection accuracy of abnormal decreases in vital capacity

**Figure 6:**
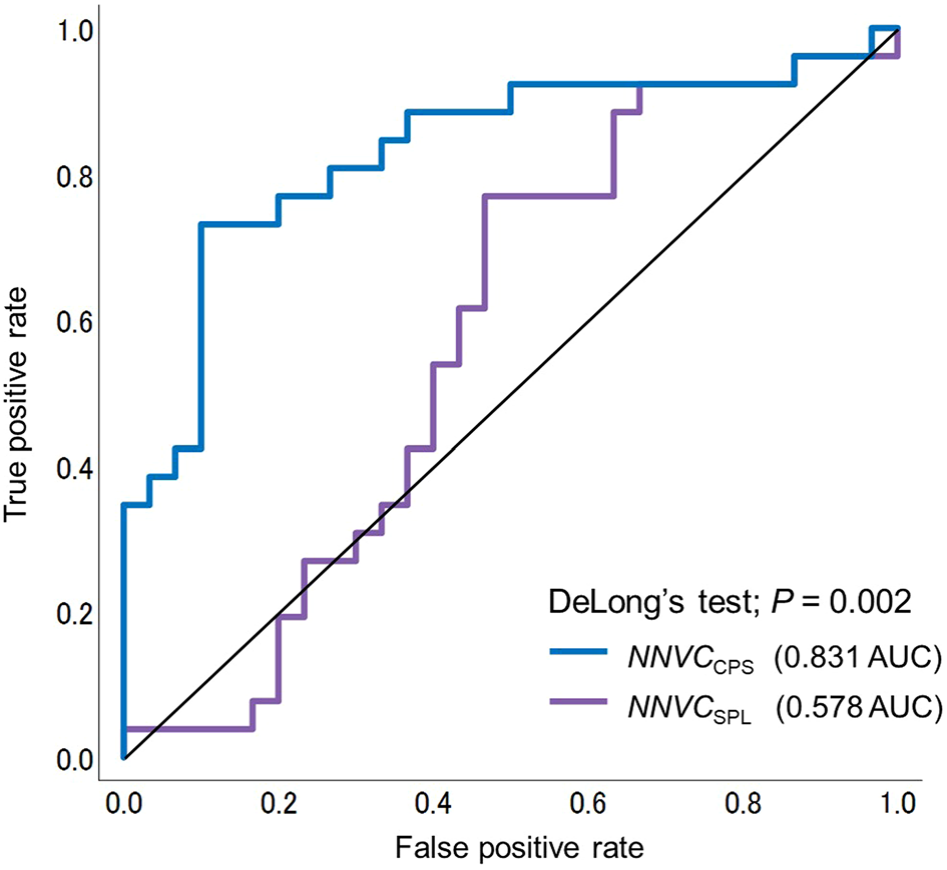
Comparison of the AUCs of *NNVC*_SPL_ and *NNVC*_CPS_ using the DeLong test. AUC; area under curve. The purple line represents the *NNVC*_SPL_. The blue line represents the *NNVC*_CPS_.

The DeLong test showed a significant difference in the AUC between the *NNVC*_SPL_ and *NNVC*_CPS_ (0.578 vs. 0.831; *p* = 0.002, respectively) (see Fig. 6). The true positive and false negative rates of the *NNVC*_CPS_ were 0.731 and 0.269, respectively.

## Discussion

This study aimed to develop a simple vital capacity evaluation method. To the best of our knowledge, this was the first study that estimated vital capacity based on cough sound. The proposed method demonstrated that an accurate vital capacity can be estimated for different individuals by using *VC*_LMS_ and the cough peak flow computed via cough sounds. In addition, we found that an abnormal decrease in vital capacity, which is associated with respiratory dysfunction, can be detected using the proposed vital capacity estimation method, with an AUC of 0.831.

First, to determine the input to the neural network-based model, the relationships between the vital capacity and different physical attributes and the cough peak flow were analysed using partial correlations. The results showed that cough peak flow, age, and height were significantly correlated with vital capacity. These relationships were consistent with those found in previous studies^14,15^. Height and age were used as independent variables for calculating the reference value of the vital capacity (*VC*_LMS_) via the LMS method. The LMS method has the advantage of reflecting age-dependent changes in respiratory function because a nonlinearly smooth fit of the vital capacity over the entire age range can be predicted^9^. Thus, *VC*_LMS_ includes information about both age and height. In addition, a previous study reported that vital capacity is related to cough peak flow^15^, which can be estimated via the cough sound pressure level, and its estimated value is *CPS*^17^. For these reasons, we hypothesized that vital capacity can be estimated by correcting the *VC*_LMS_ value using the cough peak flow computed via cough sounds or the cough sound pressure level. Thus, two neural network-based models were constructed: the first model uses *VC*_LMS_ and the cough peak flow as inputs, and the other model uses *VC*_LMS_ and the cough sound pressure level as inputs.

The experimental results showed that *NNVC*_CPS_ led to the best estimation accuracy among the three methods, and no systematic error was observed (see Fig. 3c). Furthermore, Eq. (4) incorporates an age factor, indicating that an equation that uses the cough peak flow computed via cough sounds as an input is less susceptible to the effects of aging. While the cough peak flow computed via cough sounds and cough sound pressure level are related, as shown in Eq. (4), they differ in that the age factor is included in the formula for computing the cough peak flow computed via cough sounds (Eq. 4) but not in the cough sound pressure level formula (Eq. 3). Previous studies reported that vocal fold function, a crucial factor in coughing, is negatively impacted by aging^29,30^. Thus, it is plausible that *NNVC*_CPS_ could effectively suppress the effects of aging on vocal cord function and enable the detection of decreased vital capacity. In spirometer measurements, if the difference in the vital capacity between the largest and second largest manoeuvre exceeds 0.150 L, the measurement is considered a failure, and additional trials should be performed^21^. In this study, the mean relative difference between the measured vital capacity and *NNVC*_CPS_ was 0.008 L ml (95% CI − 0.082 to 0.098), which is lower than the standard value for additional trials. A recent investigation employing dynamic chest radiography estimated the forced vital capacity, yielding a correlation coefficient of 0.86 (95% CI 0.79 to 0.90) between the measured and estimated values^31^. Similarly, a recent study examining forced vital capacity estimation via vocal analysis in patients with amyotrophic lateral sclerosis reported a correlation coefficient of 0.8, with a mean absolute error of 0.54 L^32^. Our study focused on measuring slow vital capacity, which involves slow expiration, while previous studies measured forced vital capacity, which involves fast expiration with effort. However, despite the differences in the measurement methods and participant attributes, the estimation accuracy of our proposed model is expected to be better than or at least equivalent to that of methods proposed in prior investigations. Therefore, the proposed method could have sufficient accuracy and be useful in screening tests.

We also attempted to detect abnormal decreases in vital capacity using the estimated *NNVC*_CPS_. The efficacy of this approach was confirmed, with a high AUC of 0.831 (see Fig. 6). In a previous study that discriminates restrictive impairment of lung function, spirometry values were used to calculate the difference between lung age and actual age. This method showed an AUC of 0.891^33^, which is slightly higher than the proposed model. Nonetheless, the proposed model significantly outperforms previous studies in terms of ease of measurement.

It should be noted that the false-negative rate was high, and there was a possibility of missing respiratory function decline. This suggests the effect of cases with loud cough sounds but low lung capacity. Respiratory muscle strength and cough strength have been shown to be positively correlated^15,34^. Thus, respiratory muscle strength could affect the cough sound pressure level*,* which was used to calculate the estimated cough peak flow computed via cough sounds. The estimation accuracy could be improved further to reduce the false-negative rate, such as by adding variables related to respiratory muscle strength to the input layer in the neural network.

The findings of this study suggest that the vital capacity of individual participants can be estimated with a neural network analysis approach using *VC*_LMS_ and the cough peak flow computed via cough sounds as inputs. Unlike linear models, the neural network-based model can handle nonlinear changes and was suggested to be promising for respiratory monitoring in a previous study^35^. However, the participants of this study were limited to young and elderly people without underlying diseases based on self-report. The cough peak flow computed via cough sounds used in this study, which reflects cough force, is calculated based on cough sounds. Because the cough sound may be affected by the accumulation of secretions such as sputum, narrowing of the airway due to some diseases, or inadequate closure of the glottis, it is unclear to what extent the accuracy of vital capacity estimation may be affected. Therefore, it is necessary to clarify the effects of secretions and diseases on the accuracy of vital capacity estimation in future studies. In addition, although this study estimated only vital capacity, it is necessary to estimate measures that reflect obstructive ventilation disorders, such as forced vital capacity, one-second volume, and peak flow, to construct a more comprehensive respiratory function estimation system. Moreover, to apply the proposed method to patients in home environments, it would be more convenient to measure cough sounds with a smartphone. However, it has been found that the measurement accuracy of smartphones is lower than that of in-ear microphones^17^. Thus, additional studies are needed to implement the proposed method for estimating vital capacity on smartphones. In addition, it is essential to improve the proposed method in the future so that the error between the measured vital capacity and the estimated vital capacity is minimized; then, the same cut-off reference value could be applied. Nonetheless, it should be noted that the proposed method is presented as a screening method, and a conclusive diagnosis must be based on a thorough examination at a medical institution.

## Data Availability

The data that support the findings of this study are available in the main text and from the corresponding authors upon reasonable request.
